# Montelukast in the treatment of duodenal eosinophilia in children with dyspepsia: Effect on eosinophil density and activation in relation to pharmacokinetics

**DOI:** 10.1186/1471-230X-9-32

**Published:** 2009-05-11

**Authors:** Craig A Friesen, Nancy A Neilan, Jennifer V Schurman, Debra L Taylor, Gregory L Kearns, Susan M Abdel-Rahman

**Affiliations:** 1 The Children's Mercy Hospital and Clinics, Kansas City, Missouri, USA

## Abstract

**Background:**

We have previously demonstrated the clinical efficacy of montelukast in a randomized double-blind controlled cross-over trial in patients with dyspepsia in association with duodenal eosinophilia. The mechanism of this clinical response is unknown but could involve a decrease in eosinophil density or activation.

**Methods:**

Twenty-four dyspeptic patients 8–17 years of age underwent initial blood sampling and endoscopy with biopsy. Eighteen of these patients had elevated duodenal eosinophil density and underwent repeat blood sampling and endoscopy following 21 days of therapy with montelukast (10 mg/day). The following were determined: global clinical response on a 5-point Lickert-type scale, eosinophil density utilizing H & E staining, eosinophil activation determined by degranulation indices on electron microscopy, and serum cytokine concentrations. On day 21, pharmacokinetics and duodenal mucosal drug concentrations were determined.

**Results:**

Eighty-three percent of the patients had a positive clinical response to montelukast with regard to relief of pain with 50% having a complete or nearly complete clinical response. The response was unrelated to systemic drug exposure or to mucosal drug concentration. Other than a mild decrease in eosinophil density in the second portion of the duodenum, there were no significant changes in eosinophil density, eosinophil activation, or serum cytokine concentrations following treatment with montelukast. Pre-treatment TNF-α concentration was negatively correlated with clinical response.

**Conclusion:**

The short-term clinical response to montelukast does not appear to result from changes in eosinophil density or activation. Whether the effect is mediated through specific mediators or non-inflammatory cells such as enteric nerves remains to be determined.

**Trial Registration:**

ClinicalTrials.gov; NCT00148603

## Background

Recurrent abdominal pain is a common complaint among school-age children that affects up to 15% at any given time. It represents the most common chronic pain entity in pediatric patients. These patients frequently are found to have dyspepsia defined as upper abdominal pain or discomfort. [1] In fact, eighty-seven percent of children referred to our clinic for recurrent pain have dyspepsia (either alone or in combination with irritable bowel syndrome). [2] Shaffer, et al, found dyspepsia at similar rates, being present in 73% of 154 children with recurrent abdominal pain. [3]

Duodenal eosinophilia has been associated with functional dyspepsia in adults. [4] Previously, we found duodenal mucosal eosinophilia in 71% of children undergoing endoscopy with mucosal biopsy for dyspepsia. However, eosinophil density alone may not reflect disease involvement as density does not necessarily correlate with eosinophil activation and many eosinophil-derived mediators are bioactive in a concentration-dependent fashion. [5] The degree of degranualtion may be a better indicator of the disease process rather than density. In previous investigations, we have found evidence of moderate to extensive eosinophil degranulation even in biopsies of dyspeptic children with normal mucosal eosinophil densities. [6]

Montelukast is a competitive antagonist of the cys LT_1 _receptor with an affinity that is similar to, but lower than that of leukotriene D_4_. [7] We and others previously have reported good clinical response to montelukast in patients with eosinophilic gastroenteritis. [8-11] Vanderhoof and Young reported on eight patients with dysphagia, diarrhea, and/or constipation associated with tissue eosinophilia who had prolonged remission of symptoms with montelukast therapy. [10] These experiences prompted us to undertake a double-blinded placebo-controlled cross-over trial of montelukast in 40 dyspeptic children with duodenal eosinophilia. In that study, we were able to demonstrate the superiority of montelukast as compared to placebo in the relief of pain. [11] Despite an average duration of pain of nearly 22 months prior to study enrollment, approximately one-half of the patients became pain free or nearly pain-free during the two week course of therapy with montelukast. However, the mechanism responsible for the demonstrated clinical efficacy of montelukast in dyspeptic children with duodenal eosinophilia has not been established. It is possible that the therapeutic effect might result from a lowering in eosinophil density, alteration of the eosinophil activation state, blocking leukotrienes released by eosinophils (or other cells) at their site of action, or any combination of the aforementioned effects.

Also of interest from our previous study was the finding that montelukast pharmacokinetics, and thus exposure, were different than previously observed in children receiving the drug. Specifically, the average population elimination t-1/2 for montelukast in our subjects (1.8 hours) was substantially shorter than mean values for this parameter (3.4 hours) determined from children without concurrent intestinal disease. [7,12] While the reasons for this apparent disparity are not clear, it is possible that local montelukast metabolism (i.e. in the small intestine) may vary as a consequence of disease state. Nonetheless, what remains to be determined is whether there is a link between systemic and tissue levels and whether an exposure-response relation can be established for montelukast in pediatric patients with eosinophilic duodenitis.

Thus, the objectives of the current open-label study were 1) to determine the effect of montelukast on mucosal eosinophil density and activation in pediatric patients with eosinophilic duodenitis presenting as dyspepsia; and 2) to evaluate relationships between clinical response, systemic drug exposure, local tissue drug concentration, and eosinophil density and activation.

## Methods

### Subjects

Twenty-four subjects ages 8–17 were enrolled in this study. The sample size was determined to allow for a 25% drop out rate while maintaining 80% power to detect a 33% decrease in the mean eosinophil density. Subjects were eligible for inclusion if all of the following criteria were met: 1) dyspepsia of more than two months duration, defined as persistent or recurrent pain or discomfort centered in the upper abdomen, not relieved by defecation or associated with a change in stool frequency or form; 2) scheduled for endoscopy following failure to respond to acid-reduction therapy; and 3) ability to comply with all study procedures.

Subjects were excluded if one or more of the following criteria were met: 1) previous treatment with montelukast; 2) treatment with corticosteroids or oral cromolyn sodium in the four weeks prior to enrollment; 3) any prior history, clinical signs/symptoms or biochemical evidence to suggest clinically significant alteration in hepatic or renal function; 4) exposure to drugs or natural products known to induce drug-metabolizing enzymes of the cytochrome P-450 super family, including CYP2C9 (e.g. rifampin, barbiturates) or CYP3A4 (e.g. rifampin, carbamazepine, phenobarbital, phenytoin, nevirapine, St. John's wort), or to inhibit CYP2C9 (e.g. fluconazole, fluvoxamine, paroxetine, sertraline, trimethoprim, sulfamethoxazole) or CYP3A4 (e.g. azole antifungals, grapefruit juice, clarithromycin, erythromycin, ciprofloxacin, antiretroviral agents); or 5) ingestion of Vitamin E supplements. Following endoscopy, subjects were excluded if peak duodenal bulb eosinophil density was less than 20 cells/high power field (hpf).

The study protocol was approved by the Institutional Review Board of the participating hospital. Informed parental permission and subject assent were obtained prior to initiation of study procedures.

### Study Procedures

All subjects underwent an initial endoscopy as part of a routine clinical evaluation. Standard biopsies (two from each of the following: lower one-third of the esophagus, gastric antrum, duodenal bulb, and second portion of the duodenum) were obtained from all patients for histologic evaluation and antral biopsies were obtained for rapid urease testing for *Helicobacter pylori*. Two additional antral and two additional duodenal bulb biopsies were obtained from sights adjacent to the histology biopsies for electron microscopic (EM) evaluation. These additional EM biopsies were discarded for patients who subsequently were found to have less than 20 eosinophils/hpf on duodenal bulb biopsies. At the time of endoscopy, blood was obtained for laboratory analysis as described below.

On study day 1, subjects meeting histologic criteria (more than 20 eosinophils/hpf) began treatment with 10 mg montelukast (two 5 mg tablets) orally each morning. Montelukast (Singulair^®^, 5 mg oral tablets, Merck & Co., Inc.) was supplied by the study sponsor. All subjects were prescribed age appropriate doses of ranitidine to take throughout the study. Compliance was assessed by tablet counts at the end of the treatment period.

On study day 21, treatment response was assessed in two ways:

1. Global clinical response: The evaluation employed a Likert-type scale adapted to assess change in pain by subject report. Pain was the primary symptom in all subjects.

The five pain relief grades were:

Grade 1 Worse – clinical deterioration with increasing pain intensity and/or frequency.

Grade 2 No change – no increase or decrease in pain intensity or frequency

Grade 3 Moderate improvement – partial clinical response with definite improvement in pain, but not meeting the criteria for a Grade 4 response.

Grade 4 Good – nearly complete relief of symptoms with minimal residual pain and pain not interfering with daily activities

Grade 5 Excellent – complete relief of pain

2. Histologic response: Specimens for routine histology were processed in the usual fashion and stained with hematoxylin and eosin. These were used to determine cell density for eosinophils in the esophagus, antrum, duodenal bulb, and the second portion of the duodenum. Pre- and post-treatment specimen slides were mixed together and evaluated in a blinded fashion by a single observer. Densities were determined by counting eosinophils beginning in what appeared to be the most involved area after scanning the entire specimen. Five consecutive hpfs (40×, approximately 0.8 square millimeters) were evaluated with the peak count defined as the highest count of the five fields and the mean count as the average of the five fields.

Specimens for EM evaluation were processed by the previously described methodology. [6] Pre- and post- treatment micrographs were mixed and evaluated in a blinded fashion. Eosinophil activation was evaluated by previously published methodology, with determination of the eosinophil activation index (calculated as the percentage of total granules which are activated). [6]

### Laboratory evaluation

ECP: Blood was collected by venipuncture into a 4-mL Vacutte^® ^(Greiner Bio-One, Kremsmuenster, Austria) tube without gel or anticoagulant. The tube was gently inverted 8 times then placed in a 26°C ± 1°C water bath for 1 hour ± 5 min. The tube was then centrifuged at 3,000 × g for 15 min at 4°C. Serum was harvested and stored at -80°C until analysis. Serum ECP was quantitated using a commercially available fluorescent enzyme-immunoassay platform (ImmunoCAP™, Phadia AG, Uppsala, Sweden) following the manufacturer's directions.

Cytokines: Blood was collected by venipuncture into a chilled 3-mL Vacutainer^® ^(BD, Franklin Lakes, NJ) tube containing EDTA. The tube was gently inverted 8 times then centrifuged at 3000 × g for 15 min at 4°C. Plasma was harvested and stored at -80°C until analysis. Plasma cytokines were quantitated using a commercially available multi-plex bead array kit, Cytokine 25-plex Ab Bead Kit, Hu (BioSource™, Carlsbad, California, USA) with the Luminex™ platform (Austin, Texas, USA). Cytokine analyses were performed following the manufacturer's directions with one modification. The lower limit of quantitation was extended for most of the analytes, including IL-4, IL-5, IL-8, TNF-α, MCP-1 by including an additional 3-fold serial dilution of the rehydrated standards (1:1458 dilution) in the stand curve.

### Pharmacokinetic Analysis

On day 21, all patients reported to the GI procedure room three hours prior to their endoscopy. An intravenous catheter was placed for the procedure. At 2.5 hours prior to the procedure, patients received two 5 mg montelukast tablets with up to 2 ounces of water and the time was recorded. Blood samples were obtained at 1, 2.5, and 6 hours post-dosing. Blood samples were collected in glass tubes containing sodium heparin and were mixed by manual inversion. Tubes were centrifuged (2,500 × g, 15 minutes, 4°C). Plasma was removed by aspiration and placed into polypropylene vials and stored at -80°C. At the time of endoscopy (approximately 2.5 hours post-dosing), multiple duodenal mucosal biopsies (approximate aggregate tissue yield = 0.5 gm) were obtained for determination of tissue drug concentration. Tissue samples were placed in polypropylene vials and stored at -80°C until analysis. All plasma and tissue drug concentrations were determined by the manufacturer (Merck) using a validated high performance liquid chromatographic method with fluorescence that has been used to support two previous pharmacokinetic studies of montelukast conducted in pediatric patients, both of which have successfully used the aforementioned approach for pharmacokinetic data analysis. [11,12]

The montelukast plasma concentration data from all subjects was fit using a one compartment population pharmacokinetic model with first order absorption and elimination. The pharmacokinetic model was initially parameterized using data from our previous investigation conducted in pediatric patients with functional dyspepsia. [11] Goodness of fit for the pharmacokinetic model was assessed using standard criteria (e.g., Akaike an Schwartz Information Criteria, objective function and the coefficients of variation for estimated parameters), the distribution of weighted residual estimates and the association between the observed and predicted plasma concentrations. Estimates of apparent absorption rate constant (Ka), terminal elimination rate constants (λz) and apparent volume of distribution (Vd/F) were determined for each child using the algorithms contained in the Kinetica (version 4.1.1, InnaPhase Corporation, Inc, Philadelphia, Pennsylvania, USA) software package. Estimates of apparent oral clearance (Cl/F) and area under the plasma concentration vs. time curve at steady state (AUC) were derived using the individual parameter estimates.

### Statistical Analysis

Pre- and post-treatment eosinophil densities, degranulation indices, and ECP and cytokine concentrations, respectively, were compared using paired t-tests. These tests were compared for the group as a whole and separately for patients < 12 years of age and clinical responders (≥ grade 3), respectively. Pearson correlation coefficients were determined for mucosal densities, degranulation indices, laboratory parameters, and clinical response grade. Pre-treatment laboratory parameters were compared by response grade via one-way ANOVA. Statistical analysis of pharmacokinetic data utilized a combination of approaches to assess the potential interactions between montelukast disposition and response. Relationships between continuous variables (eg., dose, ECP concentrations) were evaluated by linear and nonlinear regression techniques. A two-tailed, Student's t test was used to evaluate differences in disposition (eg. plasma vs. tissue concentrations) and response associated with gender as well as to examine exposure-response relationships for subjects classified by pain relief assessment. All statistical analyses were completed using the SSPS software package (version 15.0, SPSS Inc., Chicago, IL) and used a significance limit of α = 0.05.

## Results

### Subjects

Nineteen of the 24 subjects (79%) met the criteria of peak duodenal bulb eosinophil density of > 20/hpf. One of these patients subsequently was dropped from analysis for non-compliance with the study procedures as he had received less than one half of the medication at the 3 week re-evaluation visit. This resulted in 18 patients undergoing repeat endoscopic examination and pharmacokinetic studies. Seventy-two percent of the final sample was female. Subjects ranged in age from 9 to 16 years with a mean of 12.9 ± 2.1 years. Twenty-two percent of the patients reported a history of seasonal allergies. No patients reported food allergies but one patient had a history of crampy abdominal pain with milk ingestion. The family history was positive for allergies in 72% (13/18).

### Clinical Response

**Figure 1:**
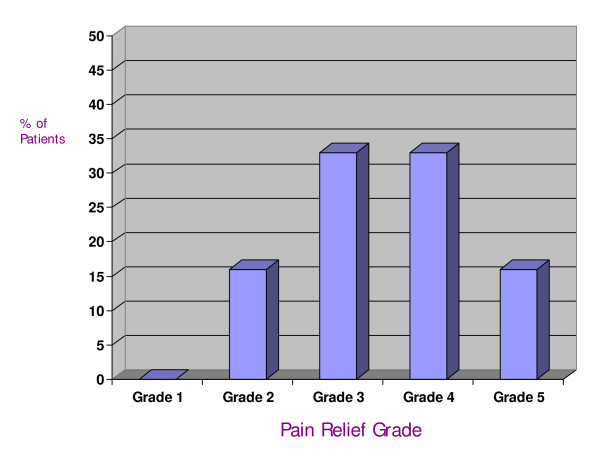
**The percentage of patients exhibiting each grade of pain relief after treatment with montelukast**.

No patients experienced deterioration while on montelukast. Three patients reported no change in pain during therapy while fifteen (83%) had a partial to complete clinical response. The percentages of subjects experiencing each level of clinical response are shown in Figure 1.

### Mucosal Response

The mean and peak eosinophil densities before and after treatment are shown in Table 1. Only a mild decrease in the peak density in the second portion of the duodenum reached significance (p = 0.04). Pre- and post-treatment densities did not differ when analyzed for clinical responders only. For subjects less than 12 years of age, the mean bulb eosinophil density was decreased post-treatment (17.2 ± 11.7 vs. 26.2 ± 15.9, p = .01) and there was a trend towards a decrease in peak bulb density (23.2 ± 11.4 vs. 36.0 ± 24.4, p = .07).

**Table 1:** Mean and Peak eosinophil densities before and after treatment with montelukast.

		Pre-treatment	Post-treatment	P Value
Esophagus:	Mean	0.24 ± 0.49	0.40 ± 1.3	NS
	Peak	0.72 ± 1.4	0.78 ± 2.1	NS
Antrum:	Mean	10.3 ± 10.1	8.9 ± 4.8	NS
	Peak	17.2 ± 14.9	16.7 ± 10.7	NS
Duodenal Bulb:	Mean	21.2 ± 11.5	18.4 ± 9.7	NS
	Peak	29.7 ± 17.1	25.8 ± 12.9	NS
2^nd ^portion duodenum:	Mean	27.9 ± 12.7	22.7 ± 9.3	NS
	Peak	39.7 ± 14.2	31.6 ± 14.9	.04

Pretreatment degranulation indices revealed mild degranulation (< 20%) in 5.6%, moderate degranulation (20–60%) in 66.7%, and extensive degranulation (> 60%) in 27.7% of patients in both the antrum and duodenal bulb. Comparing pre- and post-treatment specimens, there were no significant differences in the mean degranulation indices in either the antrum (47.8 ± 23.0 vs. 46.9 ± 24.1%) or the duodenal bulb (47.7 ± 24.3 vs. 57.1 ± 21.2%).

### Laboratory Evaluation

Eosinophil cationic protein and cytokine concentrations before and after treatment are shown in Table 2. There were no significant differences between pre- and post-treatment concentrations. Pre- and post-treatment concentrations also did not differ either for age group (<12 or ≥ 12 years) or for clinical responders.

**Table 2:** ECP and cytokine concentrations before and after treatment with montelukast.

	Pre-treatment	Post-treatment
ECP	10.6 ± 6.8	12.2 ± 9.0
IL-4	10.5 ± 5.7	11.7 ± 9.7
IL-5	1.8 ± 1.2	2.0 ± 2.1
IL-8	5.5 ± 7.8	8.5 ± 14.2
MCP-1	277.0 ± 199.7	289.3 ± 149
TNF-α	12.2 ± 15.3	10.2 ± 11.6

*all differences non-significant

Pre-treatment ECP concentration was significantly correlated with pre-treatment antral mean (r = 0.686, p = .002) and peak (r = 0.697, p = .001) eosinophil density. Post-treatment, there were no significant correlations between laboratory parameters and eosinophil density.

Only pre-treatment TNF-α concentration differed by clinical response grade (F = 6.05, p = .007) although there was a trend towards a difference for MCP-1 (F = 2.91, p = .07). Pre-treatment TNF-α was negatively correlated with clinical response grade (r = -0.519, p = .027), but positively correlated with MCP-1 (r = 0.805, p < .001) and IL8 (r = 0.570, p = .01). Other significant pre-treatment correlations for cytokines included IL4/IL5 (r = 0.916, p < .001), IL4/IL8 (r = 0.774, p < .001), IL5/IL8 (r = 0.572, p = .01), and IL8/MCP-1 (r = 0.629, p = .005).

### Pharmacokinetics

**Figure 2:**
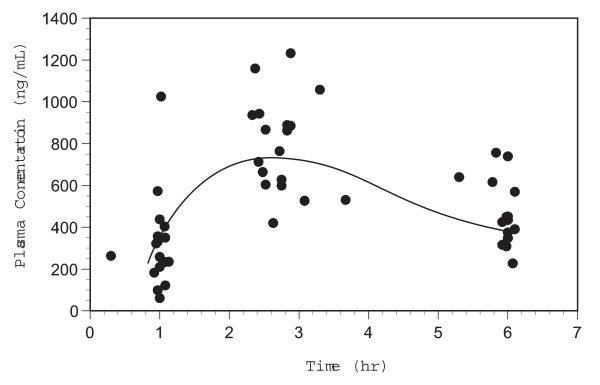
**Individual montelukast plasma concentration vs. time data**.

The composite montelukast plasma concentration vs. time data from the 18 evaluable subjects are illustrated in Figure 2. As expected, a significant degree of variability was observed in the plasma concentrations owing to a nearly three-fold difference in weight-corrected dose between the participants (range: 0.11–0.30 mg/kg). However, dose (mg/kg) accounted for only 44% of the variability observed in the 2.5 hour plasma concentration (C_2.5_) and was not predictive of the plasma concentrations observed at 1 (C_1_) and 6 hours (C_6_).

Individual pharmacokinetic parameter estimates along with relevant demographic data, are contained in Table 3. The area under the plasma concentration vs. time curve (AUC) was calculated for each child using standard equations incorporating dose, Vd/F and Kel. The average (SD) AUC was 4947.4 (1363.4) ng*hr/mL. AUC was highly correlated with post-peak plasma concentrations (r^2 ^= 0.75 and 0.83 at 2.5 and 6 hours, respectively); however, only a modest, albeit significant, relationship between dose and total body exposure (AUC) was observed (r^2 ^= 0.26, p = 0.03). In contrast, we observed no significant relationship between tissue concentrations at 2.5 hours and dose, plasma concentration, or AUC. Similarly, there were no apparent associations between age or gender and any of the montelukast pharmacokinetic parameter estimates.

**Table 3:** Demographic Data and Pharmacokinetic Parameter Estimates for Montelukast

Subject No.	Age (yr)	Dose (mg/kg)	Weight (kg)	Ka (1/hr)	Vd/F (L/kg)	Kel (1/hr)
1	13	0.188	53.3	0.532	0.08	0.334
3	14	0.166	60.2	0.577	0.06	0.399
5	10	0.293	34.1	0.609	0.14	0.475
7	14	0.125	80.1	0.545	0.09	0.354
8	13	0.272	36.7	0.555	0.11	0.386
9	9	0.22	45.4	0.498	0.11	0.293
10	11	0.305	32.8	0.553	0.13	0.396
11	14	0.14	71.2	0.554	0.10	0.375
12	11	0.305	32.8	0.507	0.14	0.354
13	16	0.176	56.9	0.544	0.14	0.401
14	15	0.187	53.6	0.535	0.13	0.369
15	15	0.169	59.1	0.570	0.10	0.396
17	10	0.173	57.7	0.538	0.11	0.355
19	11	0.152	65.6	0.546	0.08	0.353
20	13	0.223	44.8	0.548	0.13	0.394
21	15	0.115	87.3	0.565	0.11	0.395
22	14	0.19	52.6	0.605	0.08	0.424
23	15	0.123	81.2	0.538	0.09	0.345
Mean (SD)	12.9 (2.1)	0.20 (0.06)	55.9 (16.7)	0.55 (0.03)	0.11 (0.02)	0.38 (0.04)

We observed no relationship between montelukast dose or exposure (plasma concentration or AUC) and clinical responder status. Similarly we were unable to establish an association between montelukast exposure and changes in the histologic or biochemical markers of disease over the course of therapy. We did observe a positive linear relationship between C_6 _and the change in ECP between baseline and post-treatment periods (r^2 ^= 0.40, p = 0.016); however, the relevance of this finding is unclear.

## Discussion

Functional dyspepsia has been associated with duodenal eosinophilia in adults. [4] In children with dyspepsia, mucosal eosinophilia is a common finding and moderate to extensive eosinophil degranulation has been demonstrated. [6,11] Similar to previous reports, we found elevated duodenal eosinophil density (peak > 20 cells/hpf) in 79% of the patients in the current study. [11] Previously we reported moderate (20–60%) duodenal eosinophil degranulation in 65% and extensive (> 60%) degranulation in 30% of children with dyspepsia. [6] This is very similar to the degree of duodenal eosinophil degranulation in the current study which was moderate in 67% and extensive in 28%. In addition, moderate or extensive antral eosinophil degranulation was seen in 94% of patients in the current study. Findings of frequent mucosal eosinophilia and a high degree of degranulation would implicate eosinophils in the generation of dyspepsia in a subset of patients.

We previously evaluated the clinical response to montelukast in a double-blind, placebo-controlled, cross-over trial in children with dyspepsia associated with duodenal eosinophilia. [11] Montelukast demonstrated superiority to placebo in relief of pain. A positive clinical response was seen in 62.1% of patients receiving montelukast as compared to 32.4% receiving placebo. Despite a mean duration of symptoms of nearly 22 months, approximately 50% of patients became pain-free or nearly pain-free (grade 4 or 5 response) over a two-week treatment course with montelukast. The mechanism responsible for this treatment response is not known. Montelukast would have the potential to result in improved symptoms by decreasing eosinophil density, decreasing eosinophil activation, and/or blocking leukotrienes at a site of action not related to eosinophils, such as enteric nerves. The current study was undertaken to evaluate potential mechanisms of action.

In the current study, the clinical response was strikingly similar to the previous trial. A positive clinical response was seen in 83% of patients with 50% becoming pain-free or nearly pain-free (grade 4 or 5) at the end of 3 weeks on treatment. We enrolled patients with a peak duodenal bulb eosinophil density > 20 cells/hpf as we were able to demonstrate superiority to placebo in patients with 20–29 cells/hpf in the previous trial where a positive response was seen in 84% of patients (vs. 42% with placebo). In the short term, the clinical response appears to be independent of changes in eosinophil density or activation. The only change in density over treatment was a mild decrease in the peak eosinophil density in the second portion of the duodenum and there was no relationship between changes in eosinophil density and the degree of clinical response. It is possible that density might have been affected with a longer course of therapy or that a larger dose is required as has been seen with eosinophilic esophagitis. [13] While we were unable to demonstrate differences in eosinophilic activation, this may be the result of a lack of sensitivity of the method employed. While degranulation indices are general indicators of activation, they may have limited usefulness in evaluating specific pathophysiologic relationships. Eosinophils produce dozens of mediators which may be selectively released and which have varying physiologic actions. Further work is necessary to determine if montelukast alters the release of specific mediators which in turn affect the clinical response.

The mechanisms responsible for the clinical response to montelukast may involve inflammatory cells other than eosinophils or may involve leukotriene receptors on non-inflammatory cells such as enteric neurons. The clinical effect may be mediated through mast cells which have been implicated in functional gastrointestinal disorders and which contain cysteinyl-leukotriene receptors. [14] Mast cell density has been found to be increased in adults with functional dyspepsia and to be associated with delayed gastric emptying and gastric dysrhythmia in children with functional dyspepsia. [15,16] Cysteinyl-leukotrienes (cys-LTs) have been shown to alter mast cell function. For example, cys-LTs can induce IL-5 and TNF-α production in primed mast cells, an effect blocked by cys-LT inhibition. [17] Montelukast has been shown to significantly reduce the number of TNF-α positive mast cells and TNF-α concentrations in an arthritis model. [18] Montelukast's effect may also involve other inflammatory cells as montelukast has been shown to down regulate human monocyte chemotaxis induced by MCP-1. [19] The clinical effect of montelukast also could result from modulation of neuromuscular function. Leukotriene receptors are expressed on spinal sensory nerve terminals giving leukotrienes the potential to increase the sensitivity of intestinal sensory nerves during inflammation. Cys-LTs have been shown to increase depolarization and excitability of enteric neurons and to have a pro-contractile effect on esophagus, stomach, small bowel, colon, and gallbladder. [20-27] These effects appear to be mediated, in part, through cholinergic pathways. [26,27]

While others have demonstrated a decrease in serum concentrations of ECP, IL5, IL8, and TNF-α with montelukast therapy in asthma, allergic rhinitis, and cystic fibrosis, we were unable to show any decrease of these in children with dyspepsia. [28-30] Certainly the serum concentrations may not be reflective of gastrointestinal mucosal concentrations. We did, however, find a negative correlation between pre-treatment TNF-α concentrations and the degree of clinical response and there was a trend towards a negative relation between pre-treatment MCP-1 concentrations and clinical response. TNF-α, MCP-1, and IL8 concentrations were significantly correlated with each other. The clinical significance of these findings is not known. Inhaled TNF-α has been shown to increase sputum eosinophils without increasing IL4 or IL5 concentrations in asthmatics. [31] TNF-α has been found to be a vital component for chemokine generation in an eosinophil cell line and can promote a Th1 or Th2 response depending on other chemokines present in the microenvironment. [32] MCP-1 is chemotactic for eosinophils and mast cells (as well as other inflammatory cells) promoting histamine and leukotriene release. [33] High doses of montelukast in vitro have been shown to inhibit production of TNF-α and MCP-1 in peripheral blood mononuclear cells. [33] The clinical effects of montelukast may be exerted by modulation of the inflammatory response downstream from leukotrienes.

The mean pharmacokinetic parameter estimates observed in this investigation were strikingly similar to those observed in our earlier examination of montelukast in children with functional dyspepsia. [11] We continue to observe a significant degree of variability in total body exposure across this age group (range: 2636.3 to 7021.6 ng*hr/mL) which does not diminish when AUC is corrected for differences in weight-normalized dose. In contrast to our previous investigation, we were unable to detect an influence of age on montelukast pharmacokinetic parameters including distribution volume. This is likely a function of a narrower age-range in the current cohort than in our previous investigation. Finally, this investigation confirms that the terminal half-life for montelukast appears shorter in children with functional dyspepsia. As denoted in our earlier study this may reflect disease-dependent changes in presystemic bioavailability or may simply reflect errors in parameter estimation using a population-based pharmacokinetic approach. Given the shorter half-life, it is possible that different effects on inflammation might be seen with different treatment strategies such as twice daily dosing.

## Conclusion

While montelukast therapy is associated with relief of pain in children with dyspepsia who have associated duodenal eosinophilia, this response is not related to systemic drug exposure or local tissue concentrations. Also, the short-term clinical response does not appear to result from changes in tissue eosinophil density or global eosinophil activation. Whether the effect is mediated through alteration in secretion of specific mediators or an effect on non-inflammatory cells such as enteric neurons remains to be determined.

## Competing interests

This study was funded by a grant from the Merck and Co., Inc. Grants to Universities

## Authors' contributions

CF conceived of the study, participated in its design and coordination, participated in statistical analysis, and drafted the manuscript. NN participated in study design particularly related to laboratory methods and performed the EM evaluations, tissue staining, and cytokine assays, and participated in preparation of the manuscript. JS participated in the study design, statistical analysis, and preparation of the manuscript. DT participated in the design and coordination of the study. GK participated in the study design and statistical analysis. SR participated in the study design, particularly the pK analysis, performed statistical analysis, and the drafting of the manuscript. All authors read and approved the final manuscript.

## Pre-publication history

The pre-publication history for this paper can be accessed here:

http://www.biomedcentral.com/1471-230X/9/32/prepub
